# Phosphoryl Guanidines: A New Type of Nucleic Acid Analogues

**Published:** 2014

**Authors:** M. S. Kupryushkin, D. V. Pyshnyi, D. A. Stetsenko

**Affiliations:** Institute of Chemical Biology and Fundamental Medicine, Siberian Branch of the Russian Academy of Sciences, Lavrentiev Ave. 8, Novosibirsk 630090, Russia

**Keywords:** nucleic acid analogue, modified oligonucleotide, solid-phase synthesis, tetramethylguanidine, phosphoryl guanidine, phosphite

## Abstract

A new type of nucleic acid analogues with a phosphoryl guanidine group is described. Oxidation of polymer-supported dinucleoside 2-cyanoethyl phosphite by iodine in the presence of 1,1,3,3-tetramethyl guanidine yields a dinucleotide with an internucleoside tetramethyl phosphoryl guanidine (Tmg) group as the main product. The Tmg group is stable under conditions of solid-phase DNA synthesis and subsequent cleavage and deprotection with ammonia. Oligonucleotides with one or more Tmg groups bind their complementary DNA or RNA with affinity similar to that of natural oligodeoxyribonucleotides.

## DISCUSSION

Nucleic acid analogues of various structures are widely used in molecular biology and are regarded as promising therapeutic agents and probes for molecular diagnostics [1, 2]. The main impediment to the widespread application of nucleic acid derivatives for therapy is their generally poor cell uptake in the absence of specific transfection agents or delivery systems. The relative inefficiency of oligonucleotide penetration into cells can be at least partly attributed to their large net negative charge.

Only two types of nucleic acid analogues with theoretically charge-neutral backbones have been reasonably well studied over the past 20 years: peptide nucleic acids (PNA) [3] and phosphorodiamidate morpholino oligomers (PMO) [4]. Both types of analogues can bind natural DNA and RNA sequence-specifically and therefore they have found applications both in molecular biology and, in particular, in medicine as potential drugs [5, 6]. Taking this into account, the search for new nucleic acid analogues that could be used for development of oligonucleotide therapeutics capable of efficient cell penetration in the absence of transfection agents or other delivery means remains a highly relevant and challenging task.

We have shown that oxidation of 3’,5’-dithymidine- β-cyanoethyl phosphite by iodine in pyridine in the presence of 1,1,3,3-tetramethyl guanidine (TMG) yields a dinucleotide with an internucleotide tetramethyl phosphoryl guanidine group (Tmg) as the main product (*Figure A*) (similarly, oxidation of trialkylphosphite with iodine in pyridine in the presence of a primary amine yields phosphoramidates [7]). Solid-phase oligonucleotide synthesis was carried out until hexathymidylate was formed. The oligonucleotide was cleaved from the polymer using a 25% aqueous ammonia solution at room temperature for 1 h; ammonia was subsequently removed in vacuo, and the solution containing the oligonucleotide was analyzed by RP-HPLC and MALDI-TOF mass spectrometry.

**Fig.**
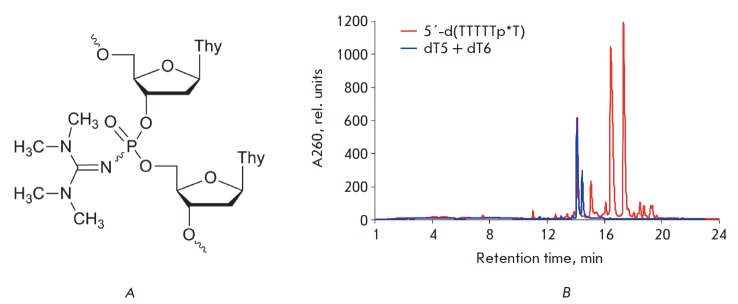
*A *– Structure of an oligonucleotide with an internucleotide tetramethyl phosphoryl guanidine group (Tmg). *B *– Elution profile of an oligonucleotide 5'-d(T_5_p*T), where p* indicates the position of the Tmg group. RP-HPLC was carried out on an Agilent 1200 HPLC system (USA) using a Zorbax SB-C18 (5 um) 4.6 × 150 mm column with a gradient of acetonitrile (0 → 40%) in 20 mM triethylammonium acetate (pH 7) for 30 min; flow rate 2 mL/min

The elution profile of the reaction mixture (*Figure B*) showed that the main product is an oligonucleotide 5'-d(T5p*T), where p* indicates the position of the Tmg group. Two main peaks with τ_R_ 16.7 and 17.6 min corresponded to the modified oligonucleotides; they were assigned to individual diastereomers resulting from chirality of the internucleotide phosphoryl guanidine group. The unmodified oligonucleotide dT6 ( τ_R_ 14.3) was also present as a by-product that most likely was formed via hydrolysis of the intermediate reactive iodophosphonium derivative by traces of moisture. The Tmg group exhibits pronounced hydrophobic properties: it is characterized by a longer retention time (τ_R_) of the Tmg group-containing oligonucleotides compared with that of the unmodified oligonucleotide. Other modified oligothymidylates up to 20 nucleotide long have been synthesized, featuring one or two Tmg groups at various positions in the oligonucleotide chain. The presence of tetramethyl phosphoryl guanidine groups in the oligonucleotides was confirmed by MALDI- TOF mass spectrometry (*Table*).

**Table:** Structures and molecular masses of phosphoryl guanidine oligonucleotide derivatives with the Tmg group

№	Oligonucleotide sequence, 5' → 3'	Molecular mass^#^		
		Calculated [M]	Experimental	
			[M + H]^+^	[M - H]^-^
1	d(TTTTTp*T)	1860.39	1860.35	1857.54
2	d(Tp*TTTTT)	1860.39	1860.34	1858.63
3	d(TCp*A)	941.80	942.17	938.97
4	d(TTTTTTTTTTTTTTTTTTTp*T)	6119.16	6121.13	6113.80
5	d(TTTTTTTTTTTp*TTTTTTTTT)	6119.16	6121.54	-
6	d(TTp*TTTTTTTTTTTTTTTTTT)	6119.16	6120.01	6114.19
7	d(TTp*TTTTTTTTTTTTTTTTTp*T)	6216.33	-	6221.11

p* indicates the position of the Tmg group. #Positive or negative ion MALDI-TOF spectra were recorded on a Bruker Reflex III Autoflex Speed mass spectrometer (Germany) using 3-hydroxypicolinic acid as a matrix.

Thermal denaturation experiments with optical registration of signal carried out at 10^-5^ M concentrations of each oligonucleotide in a 10 mM Na-cacodylate buffer (pH 7.2) containing 100 mM NaCl and 5 mM MgCl_2_ showed a relatively small effect of an isolated Tmg group at the 3’-end or in the middle of the chain on the stability of the complementary complex formed by the modified oligodeoxyribonucleotides with poly(dA) or poly(rA) templates as compared with the unmodified oligonucleotide dT_20_. The melting temperatures (*T*_m_) for the complexes formed with a poly(rA) template were 48°C for 5'-d(T_19_p*T) and 46.5°C for 5'-d(T_11_p*T9); for those formed with a poly(dA) template, the *T*_m_s were 54 and 52.5°C, respectively, which was sufficiently close to the *T*_m_ of the complexes formed by the unmodified dT_20_ oligonucleotide with the same templates (48 and 55°C, respectively). Individual d(T_19_p*T) diastereomers, which could be separated by RP-HPLC, showed only slightly different *T*_m_ values with the dC_2_A_20_C_2_ template: 45.8°C for the diastereomer with a shorter retention time and 45.1oC for the diastereomer with a longer retention time, compared with 45.1°C for the unmodified dT20 oligonucleotide. The complex of an oligonucleotide d(T11p*T9) with the modification in the middle of the chain, obtained as a mixture of diastereomers, melted at 44.7°C. These results are quite surprising, since a replacement of an internucleotide phosphate with the tetramethyl phosphoryl guanidine group leads to the substitution of a 20-atom group (including hydrogens) for one oxygen atom. The data indicated that oligodeoxyribonucleotides modified with Tmg groups can bind their complementary DNA and RNA to form stable complexes that differ only slightly in their thermal stability from the natural duplexes, a property which is a prerequisite for their therapeutic application.

To conclude, we have described a new type of nucleic acid analogues, phosphoryl guanidines [8], which is a third class of the formally charge-neutral oligonucleotide derivatives described so far in addition to known peptide nucleic acids (PNA) and morpholino oligomers (PMO). Our results indicate that oxidation of an internucleotide phosphite triester by iodine in pyridine in the presence of 1,1,3,3-tetramethylguanidine is a convenient method for obtaining oligonucleotide derivatives with the tetramethyl phosphoryl guanidine (Tmg) group. It is noteworthy that, unlike the previously described oligonucleotide analogues such as PNA or PMO, phosphoryl guanidine derivatives can be synthesized by conventional phosphoramidite chemistry using a standard DNA synthesizer. Therefore, it becomes possible to obtain various phosphoryl guanidine oligomers using a wide range of commercially available phosphoramidites, including those modified at the sugar residue and/or heterocyclic base.

We have shown that the Tmg group is stable under solid-phase oligonucleotide synthesis conditions, subsequent deprotection and cleavage from their polymer support of mixed-sequence oligodeoxyribonucleotides using standard 25% aqueous ammonia treatment at 55°C for 16 h. Oligonucleotides containing one or more Tmg groups are able to bind their complementary DNA and RNA sequences with affinity that is only slightly different from that of natural oligodeoxyribonucleotides, despite the steric bulk of the Tmg group. In our opinion, the results obtained indicate that this novel class of phosphoryl guanidine nucleic acid analogues may be quite promising for the designing of new biologically active oligonucleotide derivatives.

## Abbreviations

MALDI-TOF: matrix-assisted laser-desorption ionization time-of-flight
RP-HPLC: reverse-phased high-performance liquid chromatography
TMG: 1,1,3,3-tetramethyl guanidine
